# Senotherapeutics in Malignant Brain Cancer Therapy

**DOI:** 10.1007/s11912-025-01733-8

**Published:** 2025-12-06

**Authors:** Bernd Kaina, Markus Christmann

**Affiliations:** Institute of Toxicology, University Medical Center, Obere Zahlbacher Str. 67, Mainz, D-55131 Germany

**Keywords:** Senotherapeutics, Senopreventics, Senolytics, Senomorphics, Glioblastoma, Temozolomide, Senescence, Apoptosis

## Abstract

**Purpose of Review:**

Malignant brain cancer, the most severe form is glioblastoma (GBM), has a dismal prognosis, despite maximal resection followed by radio-chemotherapy. First line therapeutics are alkylating drugs, notably the DNA-methylating temozolomide (TMZ), administered concomitantly with radiation. Radio-chemotherapy induces not only apoptosis, but also cellular senescence in GBM cells. Senescent cells change the tumor microenvironment, cause an inflammatory response in the affected area and can be reactivated, contributing to recurrences. To eliminate therapy-induced senescent cells, senotherapeutics have gained attention. Here, we describe the pathways triggered in GBM cells leading to cellular senescence and update drugs and natural compounds acting as senolytics, senomorphics and senopreventics.

**Recent Findings:**

There is an increasing amount of data showing that temozolomide induces cellular senescence, which is even the main response of GBM cells following treatment. We outline the mechanism of senescence in glioblastoma cells and show that it rests on some unique cellular responses that may explain the low curability and aggressiveness of glioblastoma. Thus, senescent GBM cells are incompletely blocked in G2 following temozolomide treatment and undergo endoreduplications. This is presumably fostered by inactivation of CDKN2A, which is frequently mutated in gliomas.

**Summary:**

Since cellular senescence is a key event induced by temozolomide and radiation in GBM cells, it is reasonable to conclude that glioma cells cannot be completely eliminated, neither by radiation or chemotherapy alone nor in combination. Based on the data, new treatment options with senopreventics, senolytics and senostatics/senomorphics as important supportive medication during or after radiochemotherapie are discussed.

## Introduction

**Fig. 1:**
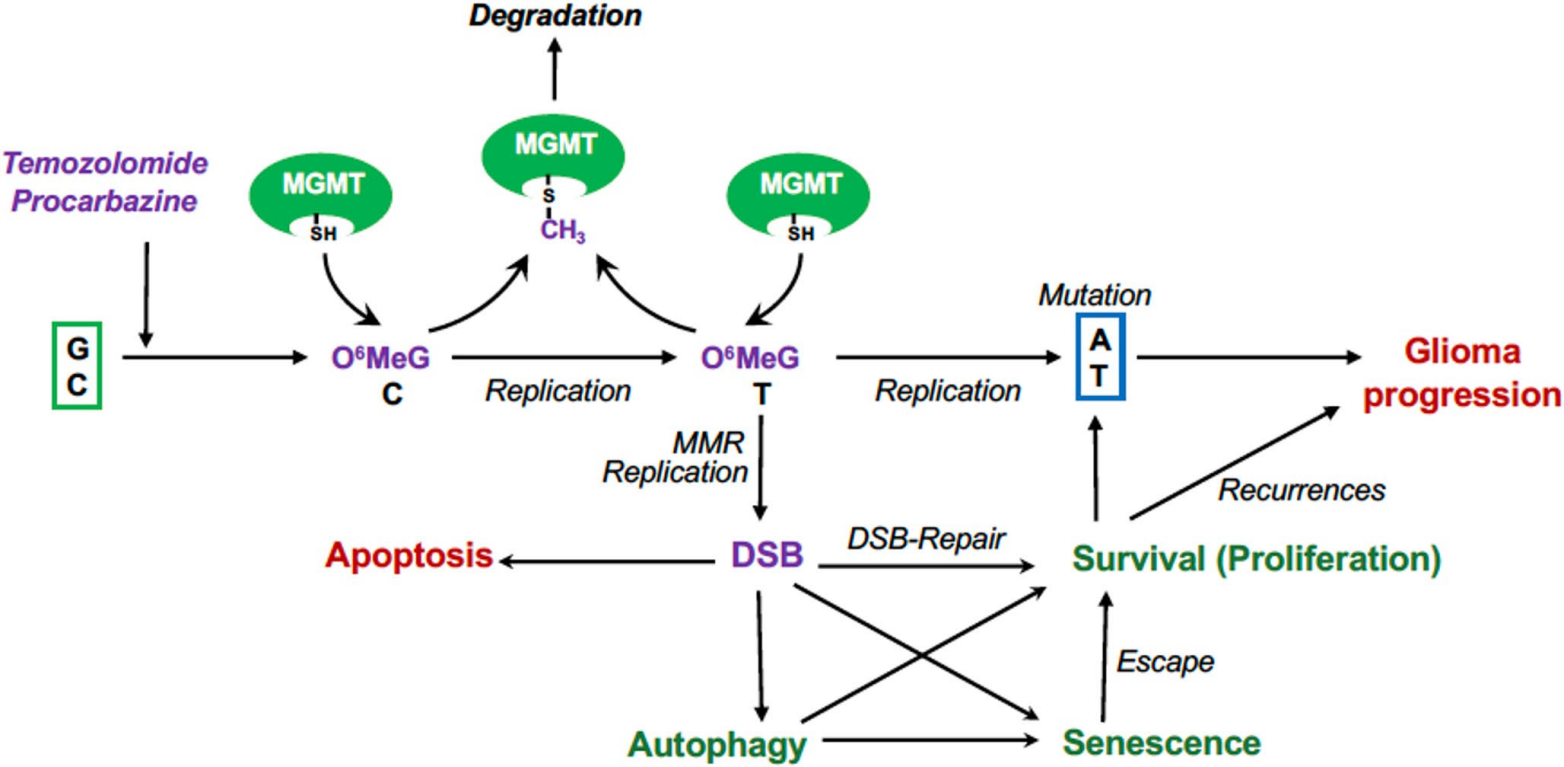
Responses triggered by temozolomide, procarbazine and other methylating drugs and defense by MGMT

DNA-alkylating agents are well-established drugs in the treatment of malignant brain tumors [1]. The group of methylating anticancer drugs comprises temozolomide (TMZ), procarbazine, dacabazine and streptozotocin. TMZ is the front-line drug for the treatment of WHO grade 3 and grade 4 gliomas, including anaplastic astrocytoma and glioblastoma [2]. Procarbazine, which is administered in the PCV scheme together with the chloroethylnitrosourea chloroethyl-cyclohexyl-nitrosourea (CCNU, Lomustine) and the mitosis inhibitor vincristine, is also frequently used in glioma therapy [3]. While procarbazine (Natulan) needs metabolic activation, TMZ spontaneously decomposes into the reactive metabolites. In both cases, carbonium ions are generated, which methylate the DNA at various sites producing, among others, the critical lesion O^6^-methylguanine [4]. The conversion of the primary DNA damage into a cytotoxic lesion needs DNA replication and mismatch repair (MMR), leading downstream to DNA double-strand breaks (DSB) and finally the activation of the DNA damage response (DDR), which triggers apoptosis, senescence and autophagy pathways (Fig. 1) [5]. TMZ is given after resection concomitantly with radiation and in subsequent adjuvant treatment cycles. Chemotherapy can extend over long periods of time since TMZ is relatively well tolerated [6], and the effects are accumulating if the primary damage is not repaired [7].

## Mechanism of Cytotoxicity

The critical primary damage O^6^MeG is, because of its mispairing properties, mutagenic (Fig. 1), but not cytotoxic per se. Cytotoxicity needs conversion of the damage into DSBs. This occurs by the mediation of MMR. A model based on reliable data states that during the first DNA replication cycle after treatment with TMZ or other DNA methylating drugs O^6^MeG/thymine mismatches are formed, which are the substrate of repetitive, futile MMR cycles. This gives rise to gaps and other distortions in the DNA that block replication in the subsequent cell cycle leading to DSBs at arrested replication forks. If not processed by MMR, mutations can arise. On the other hand, blocked replication forks and free DSBs activate the DNA damage response (DDR) pathway and induce downstream apoptosis, senescence and autophagy [8]. During cancer therapy, successful repair and escape from senescence leading to reuptake of proliferation can lead to recurrences, tumor progression and more aggressive tumor growth (Fig. 1).

**Fig. 2:**
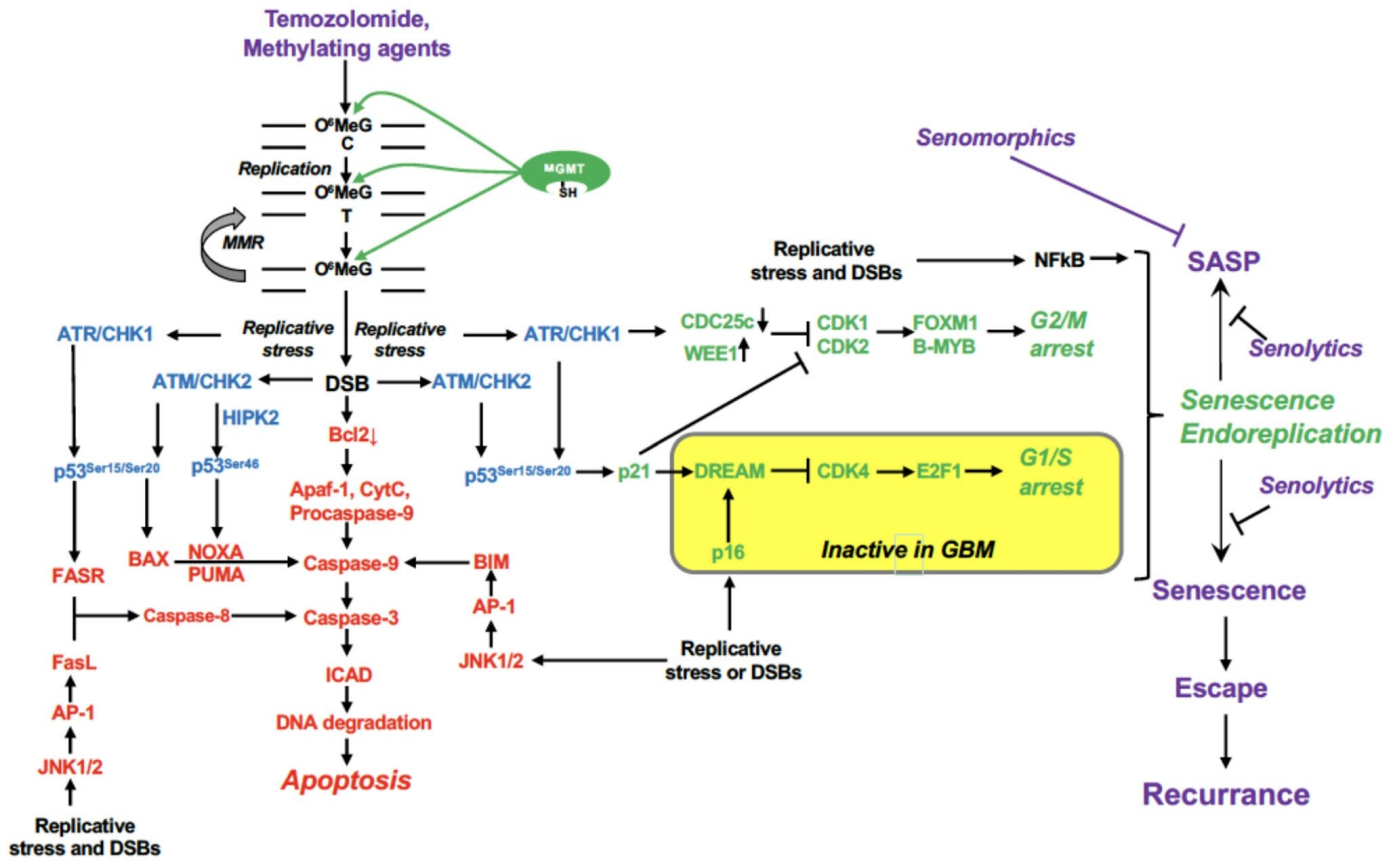
Pathways triggered by O^6^MeG in glioblastoma cells leading either to survival or death through apoptosis during therapy. It should be noted that in conserved models CDK inhibition leads to lack of Rb phosphorylation and missing activation of E2F1, which represses S-phase genes, causing arresting cells in G1. This was not the case in our study with TMZ, in which Rb was still phosphorylated and G1/S genes were normally expressed in senescent glioma cells. FOXM1 and B-MYB control G2/M genes and, therefore, inhibition of this pathway results in arrest in G2 and early mitosis [14]. The observed endoreduplication occur likely in the G2 arrested cell population that restart replication of the genome. NF-kB is a master regulator of TIS [15], which is also activated in TMZ-induced senescent glioma cells [16]. Further explanations see text

A key target of DDR kinases is p53, which regulates genes that control apoptosis pathways. It also regulates some genes involved in DNA repair causing drug resistance [9]. p53 becomes phosphorylated at various sites, which influences its activity as a transcription factor. Phosphorylation of serine 15 of the protein causes preferential activation of repair genes such as *DDB2* and *XPC* [10], while phosphorylation on serine 46 stimulates pro-apoptosis genes such as *FAS-R, BAX* and *NOXA* [11]. p53^Ser46^ is the result of stress activation of the kinase HIPK2. Under normal conditions, the kinase is inactive due to binding to SIAH1. Activation of ATM and ATR leads to phosphorylation of SIAH1, which releases HIPK2 that phosphorylates p53 [12]. We showed that glioblastoma cells treated with therapeutically relevant doses of TMZ (< 50 µM) activate the ATR/ATM-SIAH1/HIPK2-p53^Ser46^ axis and thus trigger apoptosis [8]. In addition to this signaling pathway, the Jun kinase pathway also becomes activated, which regulates both receptor and mitochondrial mediated apoptosis via the Fas ligand (FAS-L) and BIM [13]. The apoptotic signaling pathways activated by O^6^MeG upon TMZ are outlined in Fig. 2.

TMZ may also cause necrosis, which may occur under hypoxic conditions together with RT, or ferroptosis which was reported following autophagy inhibition [17] or high NRF2 expression [18]. However, these are not major traits, and there is no evidence that the pathways involved are triggered by O^6^MeG. Necroptosis rests on ATP depletion, which is caused by excessive activation of PARP1. This is a specific response following high level of DNA damage that are repaired by base excision repair (BER), causing PARP-activating BER intermediates. At clinically relevant doses this process plays no significant role.

### Mechanisms of Drug Resistance: MGMT, MMR and DSB Repair

Both O^6^-methylguanine (O^6^MeG) and O^6^-chloroethylguanine are repaired by the suicide repair O^6^-methylguanine-DNA methyltransferase (MGMT) [19]. Repair occurs in a fast and single-step reaction. In repair competent cells, O6-alkylating agents are nearly non-toxic [20], with TMZ at least in the therapeutic low dose range (<50 µM) being completely ineffective [21, 22]. On the other hand, in MGMT repair deficient cells, O^6^-alkylguanine in the tumor DNA accumulates during treatment, resulting in stepwise increase in cytotoxic effects [7]. This applies to about 40% of glioblastoma, which are MGMT promoter methylated and therefore deficient in MGMT [23], having significant impact on therapeutic outcome [24]. MGMT is otherwise expressed in all tissues, but to varying degrees: strongly in the liver, intestine, and peripheral lymphocytes, and less in the brain and very low in CD34 hematopoietic stem cells [25], which explains therapy-limiting hematotoxic side effects. The different expression levels of MGMT in the tissues and tumors as well as the inter-individual variability [26] indicates that the repair protein is subject to strong regulation on epigenetic and genetic level. Actually, hardly any other repair gene is as strongly epigenetically regulated as MGMT [27].

As outlined before, TMZ needs MMR for conversion of primary lesions into a cytotoxic damage. Therefore, enzymes involved in MMR necessarily determine the level of drug resistance. Proteins involved are MSH2, MSH6, MLH1 and PMS2. They are also regulated in cancer cells, and quantitative changes have already an impact on TMZ resistance [28–30].

During conversion of MMR-mediated secondary lesions DSBs are formed if replication meets repair intermediates (Fig. 2). These DSBs are subject to repair by homologous recombination (HR) involving Rad51, BRCA1, BRCA2 and other players, and lack or downregulation of either one of these leads to strong sensitization to TMZ [31–33]. In summary, MGMT, MMR and DSB repair by HR are the most important upstream determinants of glioma drug resistance.

## Induction of Cellular Senescence (CSEN)

While initial studies on glioblastoma cell responses were focused on cell death through apoptosis [34, 35], we and others became aware that TMZ also induces cellular senescence (CSEN) [5, 16, 36, 37] with O^6^MeG to be the primary inducing lesion [5, 16]. Apoptosis and CSEN are late events that occur in proliferating glioblastoma cells at the earliest 3 days after TMZ treatment. Senescence runs parallel to apoptosis, but with a significantly higher proportion (10–20% apoptosis versus 80–90% senescence (SA-ßGAL) measured 6 days after the onset of treatment). Under optimal conditions, 10 days after treatment >90% of the cells in the population are in the senescence stage, which is impressively visible morphologically (giant cells, large nuclei), while apoptotic cells have been successively eliminated. The remaining senescent cells are arrested in the G2 phase. Obviously, senescence is the main trait triggered by O^6^MeG in glioblastoma cells. We determined that a dose of 20 µM TMZ caused about 14,000 O^6^MeG adducts per cell, which gave rise to 7% apoptosis and 35% senescent cells in the population [21].

TMZ-induced senescent glioma cells show high levels of ROS production, oxidative DNA damage, and DSBs (visible as γH2AX foci) that are not preferentially located in telomeres [38]. Although the DSB level is high, senescent cells can still repair DSBs [38], indicating that senescence-associated DSBs are stabilized breaks not accessible to repair that were formed during senescence induction.

In line with this notion is the finding that expression of MGMT (using an inducible tet-on system) after damage induction by TMZ prevents from CSEN while expression of MGMT in senescent cells had no impact on the CSEN level [38]. This indicates that O^6^MeG is essential as a primary trigger for CSEN, but not for maintaining the senescent state. TMZ-induced senescent glioblastoma cells are also characterized by proinflammatory cytokine production, including the cytokines IL-1, IL-6, IL-8 and TNF-α [16, 39] and thus display the senescence-associated secretory phenotype (SASP).

Data on apoptosis and CSEN in vivo, following therapy of gliomas, are scare. We pursued to determine apoptosis and senescence in primary tumor specimens and corresponding recurrences. The proportion of senescent cells was significantly higher, while apoptosis was lower in recurrences compared to the primary tumor [38]. The result indicates that after radio-chemotherapy a significant proportion of tumor cells remain in the senescent stage and become visible even in the relapse, while apoptotic cells were cleared in the post-treatment period.

It should be noted that O^6^MeG is also an autophagy-inducing lesion, with the upstream pathways being identical: involvement of MMR and DSBs, and protection by MGMT. Inhibition of autophagy increased the apoptosis rate; it therefore exerts a protective function in glioblastoma cells [5]. Furthermore, we should note that ionising radiation is also a potent inducer of senescence in glioma cells [40–42]. Since radiation and TMZ are concomitantly applied for treatment, the question arises as to additive or synergistic effects in this therapeutic setting, which is worth to be studied.

Nitrosoureas such as CCNU (lomustine) are frequently applied in glioma therapy, either alone or in combination with procarbazine (PCV scheme). Whether nitrosoureas induce CSEN in GBM cells and how toxic lesions induced by the drugs are interacting is to our very best knowledge not yet explored. The same is true for tumor-treating fields (TTF), which are being used in GBM maintenance therapy, alone or together with TMZ and CCNU [43]. TTF is thought to be cytotoxic by inhibiting mitosis and DNA repair through homologous recombination [44], which is a key drug resistance mechanism. Thus, it is reasonable to hypothesize that TTF together with TMZ enhances the therapeutic response trough amelioration of cell death, but as a side effect also through the induction of CSEN.

## Molecular Mechanism of CSEN Induction in Glioblastoma Cells

It has been shown that the upstream pathway triggering apoptosis and CSEN following TMZ is identic [5]. Both endpoints need MMR causing sustained replication blockage and DSBs that are the ultimate upstream trigger. They activate the ATR-CHK1- and the ATM-CHK2- p53 axis, causing transcription of p21, which acts as inhibitor of cyclin-dependent kinases driving cells through the cell cycle. To be more precise: ATR-CHK1, which become primarily activated in the O^6^MeG pathway [45], provoke upregulation of WEE1 and downregulation of CDC25c, causing inhibition of CDK1 and CDK2 that usually activates FOXM1 and B-MYB causing a G2 arrest. At the same time, ATM-CHK2 and ATR-CHK1 activate p53 through phosphorylation of ser15 and ser20, giving rise to transcriptional activation of the CDKN1A gene and thus p21 overproduction. p21 is a CDK inhibitor blocking CDK1, CDK2, CDK4 and cyclin E through physical interaction, thus inhibiting cells in the G2 phase (Fig. 2).

An important target of p21 is the multi-repressor complex DREAM [46]. The role of DREAM in induction and maintenance of CSEN in glioblastoma cells following TMZ has been studied recently [14]. It was shown that TMZ-induced senescence does not require activation of the DREAM complex, but is bound on a G2-specific response. Thus, we showed that p21 upregulated via the ATR/ATM-p53 axis does not directly interact with CDK4, but as outlined above, with CDK1- and CDK2-cyclin E, causing abrogation of the B-Myb and FOXM1-signaling pathway, which leads to arrest of cells in the G2-phase. Interestingly, the induced G2-arrest was incomplete, presumably because of incomplete gene repression due to lack of DREAM. As a consequence, DNA synthesis in senescent cells can be resumed leading to endoreduplications. This process is preceded by reactivation of the G1/S-specific E2F1-signaling pathway, which is facilitated due to lack of functional DREAM. Incomplete DREAM activation may also explain the finding that senescent glioblastoma cells are able to repair DSBs [38] and do not show general downregulation of DNA repair, as reported for other cell systems [47–49]. The finding that CDK4 is not blocked and E2F1 still active may be taken as explanation for the incomplete G2 arrest, explaining endoreduplications, the giant cell morphology and the increase of DNA content, which is a hallmark of TMZ-induced senescent glioma cells [14].

Interestingly, the process of endoreduplication in senescent cells was subject to inhibition by the CDK4-blocking drug palbociclib, thereby stabilizing cells in the senescent stage [14]. This might be harnessed in therapy as endoreduplications and presumably also reactivation of senescent cells is prevented.

The incomplete activation of DREAM in glioblastoma cells following TMZ is very likely due to lack of CDKN2A/p16^INK4A^, which acts as specific inhibitor of CDK4/6-cyclin D [50]. Thus, up to 50% of the glioblastoma cell lines show a deletion in CDKN2a, coding for p16^INK4A^ and p14^ARF^ [51] and, according to the PanCancer atlas, 56% of glioblastoma display homozygous deletion of this gene (https://www.cbioportal.org/study/summary?id=gbm_tcga_pan_can_atlas_2018), which is the most frequently deleted gene in glioblastoma. On opposite, CDKN1A shows genomic alterations in only less than 1% of glioblastoma. Therefore, CDKN1A/p21 appears to plays a key role in drug-induced senescence in glioblastoma. This is supported by the finding that p16^INK4a^ is not involved in topisomerase I inhibitor irinotecan-induced senescence in glioblastoma cells [14].

## Can Senescent Cells Escape and Become Reactivated for proliferation?

Senescent cells exhibit a specific gene expression profile and metabolic condition and are irreversibly blocked in the cell cycle. However, there is increasing evidence that there are exceptions to the rule and that therapy-induced senescent cells (TIS) can acquire the ability to be reactivated for proliferation. Thus, reactivation of tumor cells from a senescent stage induced by etoposide and dexamethasone has been demonstrated. This was associated with weakening of the SASP, an increase in the polyploidy level and aggressive tumor growth [52]. Reactivation from the dormant stage, in which the cells can remain for a long time (months, years), is a rare event; in a lung carcinoma model, this was determined to be 1 in 10^6^ cells [53]. Although this low rate corresponds to gene mutation frequency, it is conceivable that clonal tumor regrowth can occur. If release from the dormant stage occurs months or years after radiation or chemotherapy (or biologicals inhibiting proliferation and causing CSEN), this would inevitably lead to late recurrence. In principle, one reactivated cell in the remaining senescent population would be sufficient to clonally form a tumor again. The reactivation of senescent cells for proliferation is a plausible explanation for recurrences, which can appear even years after treatment.

The finding that TMZ is an extremely efficient inducer of senescence in glioblastoma cells makes it very likely that this scenario applies to glioblastomas. Thus, it is reasonable to suppose that the induction of senescence occurs already after the first radiochemotherapy cycles, leading to a highly resistant tumor cell subpopulation (of note, TMZ needs replication and senescent cells are inherently resistant due to blocked apoptotic pathways). Furthermore, these cells likely exhibit the SASP [54], which is a hallmark of senescent glioblastoma cells after TMZ treatment [55]. Therapy-induced senescent cells secrete proinflammatory cytokines, can promote angiogenesis and modulate the immune system and thus can evade immune surveillance [56, 57]. They therefore worsen the clinical outcome. Overall, there is accumulating evidence that therapy-induced senescence plays a role in glioma treatment, recurrence and cancer progression [55, 58].

Given that the proinflammatory properties of senescent cells are a driving force in tumor progression and senescent cells can be reactivated for proliferation, three strategies are conceivable going along with genotoxic therapy: (a) prevention of induction of senescence, (b) suppression of SASP and (c) eradication of therapy-induced senescent cells. Combining senotherapeutics with radiation and chemotherapy is anticipated to enhance the effectiveness of genotoxic cancer therapies.

## Senopreventics

This strategy aims at inhibiting the induction of senescence, driving cells into the apoptotic pathway. Since senescence induction occurs *via* activation of the DDR, classical inhibitors of ATM, ATR, CHK1 and CHK2 would fit this criterium. However, these drugs are not specific, since they do not only block senescence induction, but also efficiently kill both tumor and normal cells and therefore have significant side-effects. An alternative might be a specific p21 or p16 (for p16 non-mutated tumors) inhibitors, which would force damaged cells into the cell cycle and thereby induce cell death. However specific p21 inhibitors or PROTACs that are clinically used are not available. In our previous experiments we observed that fisetin, a natural plant flavonoid, given together with TMZ enhanced the level of apoptosis and reduced senescence [59]. Similar findings were observed with artesunate, a TCM drug extracted from *Artemisia annua* L., which ameliorated the cytotoxicity of TMZ and reduced the CSEN level [60]. Thus, the natural compounds fisetin and artesunate might be considered senopreventive agents. A recent study showed that astemizole, a hERG/Eag1 K⁺ channel blocker, administered together with TMZ ameliorated apoptosis and reduced the yield of CSEN [61], thus being effective as senopreventics.

## Senolytics

These drugs (compiled in Table 1) are defined being agents that specifically induce death of senescent, but not proliferating or resting non-senescent (differentiated) cells. The discovery of senolytic drugs was based on the observation that senescent cells are resistant to apoptosis, due to upregulation of specific senescent-cell anti-apoptotic pathways (SCAP) caused by senescence-associated mitochondrial dysfunction (SAMD) [64, 65]. Although it’s early days and evidence is limited, senolytics targeting these SCAP have been tested in several clinical trials on aging and geriatric syndroms [66]. Among these, especially inhibitors of the antiapoptotic members of the BCL-2 family (BCL-2, BCL-W and BCL-X_L_) induce mitochondrial-mediated apoptosis in senescent cells. Thus, ABT-737 and ABT-263 (navitoclax) act predominantly by inhibiting BCL-2, and A-1331852 and A-1155463 by inhibiting BCL-W and BCL-X_L_. In vitro, navitoclax was shown to be senolytic in HUVECs, IMR90 human lung fibroblasts and murine embryonic fibroblasts, but not human primary preadipocytes [67]. The compounds A1331852 and A1155463 proved to be senolytic also in HUVECs and IMR90 cells, but not in preadipocytes [68]. This data shows the existance of cell type specificities. In patient-derived GBM lines triggered into senescence by radiation and TMZ, navitoclax, A1331852 and A1155463 showed senolytic activity [63]. In vivo, treatment with A1331852 eliminated senescent cholangiocytes and thereby reduced liver fibrosis in mice [69].

**Table 1:** (A) senolytic drugs positively tested in TMZ-induced senescent GBM cell lines [62]. (B) senolytic drugs positively tested in radiation- and TMZ-induced senescent GBM cell lines [63]. (C) senolytics not yet tested in GBM cells (see text)

Senolytic agent Mechanism	
A	
ABT-737	Bcl-2/Bcl-xL/Bcl-w inhibitor
ABT-263 (Navitoclax)	Bcl-2/Bcl-xL inhibitor
Chloroquine	Autophagy inhibitor
PX-866	PI3K/autophagy inhibitor
BV-6	c-IAP/XIAP inhibitor
AZD1390	DNA damage response (ATM) inhibitor
VE-821	DNA damage response (ATR) inhibitor
Fisetin	Flavonoid; BCL-xL pathway modulation
Artesunate	Generates ROS; anti-malarial derivative
Curcumin	Anti-oxidative/apoptosis modulator
***B***	
A1331852	Bcl-xL inhibitor
A1155463	Bcl-xL inhibitor
ABT-263 (Navitoclax)	Bcl-2/Bcl-xL inhibitor
***C***	
Piperlongumine	Unknown
Gingerone A	Unknown

Previously, we demonstrated that targeting either c-IAP1 and c-IAP2 using BV6, or Bcl-2 using venetoclax can eliminate senescent cells following TMZ [54]. BV6 was also effective in killing senescent GBM cells triggered by the anticancer drug irinotecan [70].

ABT-737 was originally shown in vivo to eliminate senescent cells induced by DNA damage in the lung as well as through activation of p53 in a transgenic p14^ARF^ mouse model [71]. Oral administration of ABT-263 to either sublethal irradiated or normally aged mice depleted senescent bone marrow hematopoietic stem cells and senescent muscle stem cells and thus rejuvenated aged mice [72]. Also, proteasomal degradation of BCL-2 provoked by the curcumin analog EF24 was shown to mediate senolytic effects [73] and the senolytic activity of cardiac glycosides like ouabain is, at least partially, caused by interfering with SCAP, activating the proapoptotic BCL-2 family member NOXA [74].

In glioblastoma cells some of the compounds were tested. In our hands, ABT-737 induced a robust and reliable senolytic response. However, the compound is not yet in the clinic, while ABT-263 (navitoclax), which was effective in eliminating senescent GBM cells [62], is clinically approved and being used for the treatment of some forms of leukemia, myelofibrosis and some solid cancers. The derivative venetoclax is more selective in inhibiting Bcl-2, does not show side effects like thrombocytopenia and thus is going to replace navitoclax [75]. Venetoclax was not yet tested on gliomas.

Some senolytics act by interfering with cell death-related signaling cascades involving tyrosine kinases, or the target of rapamycin (mTOR) pathway. This seem to be very effective strategies. Thus, the multi-kinase inhibitor dasatinib was shown to be senolytic in different cell systems, and the combination with quercetin showed superior senolytic activity both in vitro and in vivo, eliminating senescent cells in chronologically aged, radiation-exposed, and progeroid Ercc1^-/∆^ mice. In old mice, this senolytic effect improved the cardiac function and delayed age-related symptoms like osteoporosis [76].

Using dasatinib and quercetin, a direct involvement of senescent cells in aging related disease and the effectiveness of their elimination for health was shown in vivo. Transplanting senescent cells into young mice induced senescence in the host tissue and led to persistent physical dysfunction. Transplanting senescent cells in old mice showed similar effects and reduced the lifespan of the animals. Under these conditions, combined treatment with dasatinib and quercetin eliminated senescent cells, reduced the physical dysfunction and increased the overall survival [77]. Also, in clinical phase I studies performed in patients with diabetic kidney disease [78] and idiopathic pulmonary disease [79], the combination of dasatinib and quercetin reduced the amount of p16^INK4a^/SA-β-gal positive cells. The combination of dasatinib and quercetin has also been shown to be senolytic in senescencent lung fibroblasts during idiopathic pulmonary fibrosis in mice [80], as well as in the medial layer of aorta from aged and hypercholesterolemic mice, which improved vasomotor function and blood flow in the animals [81]. Taken together, the combination of dasatinib and quercetin appears to be the most effective strategy in senolytic therapy. As dasatinib is well established as to therapeutic dose and side effects, its use as a repurposed drug should facilitate clinical trials with glioma patients. Of note, senolytic therapy means short-interval treatment, for which unwished side effects are negligible.

Of special interest are natural compounds with senolytic activity (Table 1). Quercetin and fisetin belong to this group. They are natural flavonoids present in fruits and vegetables. Quercetin was shown to be cytotoxic for GBM cells by inhibiting the AXL/IL-6/STAT3 signaling pathway without affecting Akt or MAPK [14, 48]. Quercetin notably in combination with resveratrol is pro-apoptotic and induces a senescence-like growth arrest in glioma cells [82]. In human lung fibroblasts treated with doxorubicin quercetin was effective in triggering senescent cell death [83]. Although the cytotoxic and senolytic activity of quercetin is well described, robust studies on the senolytic activity of the flavonoid given alone or in combination with dasatinib to GBM cells are not yet available.

Fisetin was shown to be senolytic in senescent human vascular endothelial cells (HUVECs), but not in human lung fibroblasts and primary human preadipocytes [68], indicating cell specificity. In vivo, fisetin was reported to exert marked effects. Thus, treatment of progeroid mice with fisetin reduced senescence markers in multiple tissues, reduced age-related pathology, and extended the median and maximum lifespan [84]. In our studies with TMZ-induced GBM cells, fisetin was clearly effective in inducing apoptosis in senescent cells, confirming its senolytic activity [62]. Interestingly, fisetin at high dose level (40–80 µM) exerts genotoxic activity, inducing DNA damage (DSBs) and p53 activation. Furthermore it enhanced the cytotoxic effects of alkylating agents [59]. Therefore, the compound might be considered a reasonable supplement in GBM therapy.

Another senolytic compound is piperlongumine, a natural ingredient of the long pepper (Piper longum), which has been shown to eliminate human fibroblasts upon senescence-induction by radiation, replicative exhaustion, or ectopic expression of Ras [85]. The mechanism underlying the senolytic activity is not yet clear.

A screening study with several plant extracts and IR-induced human fibroblasts reveald ginger extract causing selective CSEN death. The active agent was identified gingerone A, which is able to elicit an apoptotic program in senescent cells [86].

Finally, our own screening study on TMZ-induced senescent GBM cells performed under standardized conditions should be summarized. It reveald that ABT-737, navitoclax, chloroquine, ATMi, ATRi, BV-6, PX-866 and the natural compounds fisetin and artesunate exhibit senolytic activity, inducing death in senescent GBM cells clearly more effectively than in the proliferating cell population [62]. Similar to fisetin, artesunate exhibited genotoxic activity, inducing oxidative DNA damage and DSBs [87, 88]. The drug seems to exert pleiotropic effects as it inhibits HR and ameliorates the therapeutic effect of TMZ upon coadministration [60]. The cytotoxicity on CSEN cells might be speculated to be due to excessive mitochondrial damage through sustained ROS formation.

In the study refered to above, no specific effect on CSEN was observed by inhibition of CHK1/CHK2, p21, NF-kB, Rad51 and PARP. We concluded that these factors neither play a critical role in maintaining TMZ-induced CSEN nor can their inhibitors be considered as senolytics [62]. It should be noted that IR and CCNU, which are usually applied alone or in combination with TMZ or procarbazine (PCV scheme) were ineffective in killing senescent GBM cells [62].

There are other senolytic mechanisms reported. Thus, the histone deacetylases (HDAC) inhibitor panobinostat has been described as senolytic, which eliminates senescent cells that were accumulating during standard chemotherapy in lung cancer patients [89]. The FOXO4 peptide (proxofim), which perturbs the FOXO4/p53 interaction, induced apoptosis in senescent cells and restored fitness, fur density, and renal function in premature aging *Xpd*^TTD/TTD^ and naturally aged mice [90]. The compunds have not yet been tested on GBM model systems or in clinical settings.

Besides inhibitors of targets involved in the maintenance of CSEN, other senolytic strategies are under development. Thus, the increased SA-β-gal activity of senescent cells was harnessed to deliver cytotoxic agents or senolytic drugs coated with galacto-oligosaccharide nanoparticles into lysosomes of senescent cells [91–93]. Another strategy is based on the finding that senescent cells are subjected to immunosurveillance [94, 95]. Based on the assumption that senescent cells might accumulate during aging due to declined immune response, it is currently tested whether restoration or activation of the immune system could specifically eliminate senescent cells (for review see [96]).

## Senomorphics/senostatics

Senomorphics and senostatics are compounds that suppress the senescence-associated secretory phenotype (SASP) without causing death of senescent cells. The terms are often used synonymously. However, it would be prudent to reserve the term “senostatics” for treatments that aim to stabilize the senescent state and prevent the escape of senescent cells. An example is the CDK4 inhibitor palbociclib, which we have shown to stabilize TMZ-induced senescent cells in G2 [14].

The SASP is characterized by secretion of multiple immune factors, including interleukins, chemokines, growth factors and matrix metalloproteinases [56, 97]. Important SASP factors are the interleukins IL-6 and IL-8 as they seem to modulate CSEN [98]. Thus, depletion of IL-6 abolished oncogene-induced senescence [97] and IL-8 increased ROS production and DNA damage [99]. Beside IL-6 and IL-8, IL-1α has been proposed as a general autocrine regulator of SASP [100]. The SASP can play both a tumor-promoting and a tumor-suppressing role [101]. As tumor-suppressing mechanism, SASP can reinforce the growth arrest by increasing ROS production and enhancing DDR, thus stabilizing the CSEN phenotype [97, 99]. In addition, SASP induces an inflammatory response and activates immune cells which can eliminate senescent tumor cells [102, 103]. On the other hand, SASP factors also act as potent tumor promoters, driving tumorigenesis. As an example, SASP can enhance the proliferation of neoplastic epithelial cells [104], and promotes EMT [105] as well as tumor growth in vivo [106, 107]. Of note, SASP factors do not only promote cancer, but also trigger multiple chronic and degenerative aging-related pathologies like neurodegenerative diseases and diabetes [98, 108].

Having said this, it follows that inhibitors of factors involved in SASP are useful for reducing the deleterious effects of senescent cells. Senomorphic activity was shown for several agents like the IL-1R antagonist anakinra, the IL1 antagonizing antibodies canakinumab and rilonacept, the THF antagonizing compounds etanercept and infliximab, as well as the IL-6R antagonizing antibody tocilizumab and the IL-6 antagonizing antibody siltuximab (for further reading see [109]).

An important factor involved in the regulation of the SASP is NF-κB. Thus, several compounds, which interfere with NF‐κB signaling (metformin, apigenin, kaempferol and BAY 11–7082) possess senomorphic activity [109]. The NF-κB pathway shows crosstalk with p38K since the p38K inhibitor SB203580 reduced NF-κB transcriptional activity and subsequently the transcription and secretion of IL-6 and IL8 in normal human fibroblasts, which underwent senescence following IR or oncogenic H‐RAS^V12^ expression [110].

An important regulator of cytokine production is the JAK/STAT (Janus kinase/signal transducer and activator of transcription) pathway. In line with this, the JAK inhibitor ruxolitinib suppressed the mRNA levels of the SASP components IL-6, IL-8, and MCP-1 in human primary IR-induced senescent cells [111].

Already more than 10 years ago, it was shown that rapamycin, an inhibitor of the mTOR pathway, was able to delay several ageing-related dysfunctions and increase the lifespan of mice [112]. Newer studies showed that rapamycin significantly decreased IL6 secretion in normal human fibroblasts and immortal, but non-tumorigenic human breast epithelial cell lines, in which senescence was induced by ionizing radiation and other stimuli [113]. However, it did not reverse cellular senescence, but repressed the ability of senescent cells to stimulate cell proliferation and tumorigenesis in mice. Mechanistically, rapamycin suppresses the translation of the membrane-bound cytokine IL-1A, which normally stimulates NF-κB transcriptional activity [113]. Delivery of rapamycin to senescent cells via CD9 monoclonal antibody-conjugated lactose-wrapped calcium carbonate nanoparticles induced anti-senescence effects (reduced β-galactosidase and reduced p53/p21/CD9/cyclin D1 expression) in old human dermal fibroblasts [114].

Although we know much about SASP and senomorphics, our knowledge regarding translation to brain cancers is very limited. These gaps in knowledge justify intensive research in this area addressing several questions: Do gliomas exhibit SASP in vivo? Which cytokines are released. Are there differences in CSEN level and SASP in tumor grading? Is SASP related in any way to MGMT, MMR and IDH1/2 status? Does the administration of senomorphics improve treatment and impacts patient's survival?

## Conclusions and Summary

The signaling pathways triggered by TMZ and other methylanting agents in GBM cells are well described. Although they provoke cell death through apoptosis, induction of CSEN is clearly the major trait. CSEN cells are inherently resistant to genotoxic therapies. Therefore, complete elimination of the tumor through radiation and chemotherapy is strictly impossible.

Therapy-induced senescent GBM cells are arrested in G2 with incomplete DNA replication inhibition, resulting in endoreduplications. Whether this facilitates release of senescent cells into proliferating state needs to be clarified. It is clear, however, that senescent cells cannot only be reactivated, but also drive tumor progress through SASP.

In view of this, it is appropriate to include into the genotoxic therapy with radiation, TMZ, procarbazine and CCNU supportive treatments with senopreventics, senolytics and senomorphics administered either comcomitantly (senopreventics), in a hit-and-run fashion (senolytics) or during maintenance therapy (senomorphics).

Senolytics active in GBM cells include natural substances such as fisetin, quercetin and artemisinin (artesunate). The natural compounds deserve special attention. Fisetin is a flavonoid and polyphenol found in smoke tree (fiset wood), various fruits (apples, strawberries, grapes, persimmons) and vegetables (cucumbers, onions). Like artesunate, it is genotoxic in glioblastoma cells at high doses. Fisetin has been shown to be an effective senolytic agent in various tumor cell systems [57] and in mice, oral administration of fisetin led to an extension of lifespan, most likely by selectively killing aged cells [58]. Fisetin has therefore gained popularity as a dietary supplement and senolytic agent in healthy individuals. 

Overall, natural senotherapeutics are well tolerated and gained popularity as dietary supplements taken by a wide range of people. It is possible that these natural substances, together with the anticancer effect of curcumin, are effective in adjuvant therapy. Chloroquine has also been shown to exert a senolytic effect on glioblastoma cells. Case reports allow the conclusion that these natural senolytics have no adverse side effects (unpublished observations and [115]). Clinical studies will have to provide information on their effectiveness. Importantly, a first clinical phase I trial, which started in July 2025, will test the safety, side effects and effectiveness of combinations treatment of glioma residual disease using dasatinib, quercetin, fisetin and TMZ (NCT07025226, https://clinicaltrials.gov/study/NCT07025226).

In addition to natural substances, well-known pharmaceuticals are also coming into focus, such as dasatinib, simvastatin and metformin. Simvastatin has been shown to enhance TMZ-induced apoptosis by inhibiting autophagy [59] and, together with metformin, inhibits glioblastoma growth through a senolytic effect, which has been shown in vitro, in animal models and in initial clinical studies [60].

## Key References

- Kandhaya-Pillai, R.; Miro-Mur, F.; Alijotas-Reig, J.; Tchkonia, T.; Schwartz, S.; Kirkland, J. L.; Oshima, J., Key elements of cellular senescence involve transcriptional repression of mitotic and DNA repair genes through the p53-p16/RB-E2F-DREAM complex. *Aging *2023, 15, (10), 4012-4034.
  - ○ This paper of importance provides evidence that the DREAM complex is a general repressor in senescent cells, downregulating also DNA repair genes.
- Di Micco, R.; Krizhanovsky, V.; Baker, D.; d'Adda di Fagagna, F., Cellular senescence in ageing: from mechanisms to therapeutic opportunities. *Nature reviews. Molecular cell biology *2021, 22, (2), 75–95.
  - ○ This review is a comprehensive review of importance on senescence.
- Bujarrabal-Dueso, A.; Sendtner, G.; Meyer, D. H.; Chatzinikolaou, G.; Stratigi, K.; Garinis, G. A.; Schumacher, B., The DREAM complex functions as conserved master regulator of somatic DNA-repair capacities. *Nat Struct Mol Biol*2023, 30, (4), 475–488.
  - ○ This is a highly important paper describing the role of DREAM as master regulator of DNA repair is senescent cells.
- Schmidt, A.; Allmann, S.; Schwarzenbach, C.; Snyder, P.; Chen, J. X.; Nagel, G.; Schoneis, A.; Rasenberger, B.; Beli, P.; Loewer, A.; Hofmann, T. G.; Tomicic, M. T.; Christmann, M., The p21CIP1-CDK4-DREAM axis is a master regulator of genotoxic stress-induced cellular senescence. *Nucleic Acids Res *2024, 52, (12), 6945-6963.
  - ○ This paper describes the p21-CDK4-DREAM axis to be activated after genotoxic stress induced by environmental carcinogens like benzo(a)pyrene.
- Schwarzenbach, C.; Rinke, J.; Vilar, J. B.; Sallbach, J.; Tatsch, L.; Schmidt, A.; Schoneis, A.; Rasenberger, B.; Kaina, B.; Tomicic, M. T.; Christmann, M., Therapy-induced senescence of glioblastoma cells is determined by the p21(CIP1)-CDK1/2 axis and does not require activation of DREAM. *Cell Death Dis *2025, 16, (1), 357.
  - ○ This paper of importance shows that in glioblastoma cells defective in p16 the p21-CDK1,2 axis regulates senescence and DREAM without the activation of functional DREAM.

## Data Availability

No datasets were generated or analysed during the current study.
